# A deep learning-based model using hybrid feature extraction approach for consumer sentiment analysis

**DOI:** 10.1186/s40537-022-00680-6

**Published:** 2023-01-13

**Authors:** Gagandeep Kaur, Amit Sharma

**Affiliations:** 1 Research Scholar at Department of CSE, Lovely Professional University, Punjab, India; 2 Symbiosis Institute of Technology (SIT), Affiliated to Symbiosis International (Deemed University), Pune, India; 3 Lovely Professional University, Punjab, India

**Keywords:** Sentiment analysis, Aspect feature extraction, Deep learning, LSTM, Hybrid features, Consumer review summarization

## Abstract

There is an exponential growth in textual content generation every day in today's world. In-app messaging such as Telegram and WhatsApp, social media websites such as Instagram and Facebook, e-commerce websites like Amazon, Google searches, news publishing websites, and a variety of additional sources are the possible suppliers. Every instant, all these sources produce massive amounts of text data. The interpretation of such data can help business owners analyze the social outlook of their product, brand, or service and take necessary steps. The development of a consumer review summarization model using Natural Language Processing (NLP) techniques and Long short-term memory (LSTM) to present summarized data and help businesses obtain substantial insights into their consumers' behavior and choices is the topic of this research. A hybrid approach for analyzing sentiments is presented in this paper. The process comprises pre-processing, feature extraction, and sentiment classification. Using NLP techniques, the pre-processing stage eliminates the undesirable data from input text reviews. For extracting the features effectively, a hybrid method comprising review-related features and aspect-related features has been introduced for constructing the distinctive hybrid feature vector corresponding to each review. The sentiment classification is performed using the deep learning classifier LSTM. We experimentally evaluated the proposed model using three different research datasets. The model achieves the average precision, average recall, and average F1-score of 94.46%, 91.63%, and 92.81%, respectively.

## Introduction

The Covid-19 threat has led to an exponential rise in online purchases of food, electronics, clothes, etc. [7, 23]. As a result, customers have posted numerous reviews online. These reviews give valuable insights into the customer's demands and fulfillment. Corporate sectors like restaurants and e-commerce websites can utilize such reviews as a tool to increase their efficiency by analytically tracking product/service-specific reviews. In addition, the reviews can be used to analyze competitors exhaustively. Nonetheless, it becomes difficult to analyze reviews due to the large volume, diversity, and momentum with which they are persistently posted online.

The approach of analyzing the behavior of emotions, opinions, and feelings for a specific entity or subject that is expressed in the form of text is known as opinion mining or sentiment analysis. The accomplishment of sentiment analysis can be achieved at the word, phrase, sentence & document level [47]. Performing it at the word level incorporates the determination of the opinion of an individual about the products, services, or their aspects. Consider the sentence, “I am happy with Samsung’s M32 battery performance” indicates a positive opinion for the battery aspect of one of the models of Samsung. The accomplishment of SA at the phrase level helps in entailing the detection of the opinion of multi-word expressions. Next, sentence-level SA involves the uncovering of the overall emotion of the sentence. Lastly, document-level SA involves the determination of the overall opinion of one or more sentences using average or weighted methods [33, 43].

The most widely employed approaches to perform sentiment analysis include the Machine Learning/Deep Learning based method, lexicon-based method, and hybrid methodology [27, 27, 28, 28, 34, 39, 43, 49]. The lexicon-based methodology makes use of a dictionary of words marked by sentiment to find out the overall opinion of a sentence. Sentiment ratings are consistently combined with additional rules to lessen the count of sentences containing sarcasm, dependent clauses, or negations. Tokenization, Stemming, Part-of-Speech (PoS) tagging, and lexicons are among the NLP techniques which are contained within the rules. The sentiment analysis systems which are lexicon-based are believed to be modest as the successive integration of words is not taken into consideration. Lexicon-based systems require frequent fine-tuning and maintenance, which further increases the efforts required for implementation.

In machine learning or deep learning-based approaches, the dataset is separated into training and testing datasets. A training data set is used during the training process so that the model discovers to associate the specific input text to the conforming yield to learn the documents. The testing dataset is used during the prediction process where the transformation of hidden textual inputs into feature vectors is done using a feature extractor. The model is then fed these vectors, which generate prediction tags for the corresponding vectors. The hybrid approach for sentiment analysis is the implementation of a Lexicon-based approach combined with a machine learning-based approach [24].

All these techniques have demonstrated remarkable accomplishment in formal language, conventional text sources, and in well-specified fields where pre-labeled data is accessible for training or the lexicon coverage includes those words that convey specific sentiments within a corpus. But these approaches are incompetent to match up to the large quantity, diversity, and momentum with which the unstructured data is constantly posted online.

The performance of sentiment analysis approaches utilizing machine learning techniques has recently improved because of the introduction of various kinds of feature extraction methods that are essential to produce accurate results [40, 45]. As part of the feature extraction phase, many challenges exist, like finding unreliable and ambiguous features for classifying the sentiments. It is, therefore, necessary to design a hybrid model of feature extraction for an accurate outcome that will overcome such challenges. Aside from sentence or review-specific features, specific products/services, their aspects, and emoticons are also required to be deemed for accurate and clear extraction of features.

For instance, contemplate the review “Although this mobile phone is too heavy, it is little cheap”, includes implicit aspects: weight (implied by the term heavy), price (implied by the term cheap), and sentiment-bearing word relations. In general, sentiment appears neutral, however, aspects have both negative and positive polarities. Further, these implicit aspects are poorly described and are not articulated using standard terms and definitions. This issue can be solved by grouping very similar aspects into attributes and then analyzing them with the Aspect-based Sentiment Analysis (ABSA) approach [9, 13, 18, 19]. In recent years, a wide range of methodological approaches have been suggested for ABSA, but a more sophisticated mechanism is needed to capture the relationship existing between implicit words, detect similar aspects, and handle ambiguities and special terms.

The functionality of the model suggested in this paper is divided into two stages: review mining and review summarization. Each user's reviews are categorized as either negative, positive, or neutral in review mining. In review summarization, a concise summary for corresponding reviews gets automatically formulated based on the results of the review mining step. The scope of this work is restricted to the development of a reliable, accurate, and robust review mining model (also called sentiment analysis model) that can further result in building an effective consumer review summarization model.

In this research, we present a unique sentiment analysis technique based on the robust hybrid analysis of sentiments which is used to solve the challenge of obtaining efficient review mining results for consumer review summarization. To begin, a straightforward and efficient pre-processing technique is used to eliminate stop words, digits, meaningless words, and other undesirable elements. Then, the preprocessed reviews are given as input to the robust and efficient hybrid feature engineering technique, which combines review-related features and aspect-related features and forms a hybrid feature vector (HFV). The hybrid feature extraction methodology suggested in this paper can deal with ambiguous and inconsistent sentences as well while performing sentiment analysis.

## Related work

The method proposed in this work is focused on analyzing sentiments efficiently, thus this segment comprises some recent studies categorized into three fields: Sentiment Analysis, Aspect-Based Sentiment Analysis (ABSA), and Deep Learning-based SA methods. Towards the end of this section, the motivation and contribution of this research are also discussed.

### Sentiment analysis (SA) techniques

This section focuses on the study of several recent works carried out in the sphere of sentiment analysis [6, 11, 15, 17, 22, 36, 38, 48, 51, 53].

The SentiDiff algorithm proposed by Wang et al. [51] combines textual information with sentiment diffusion patterns to enhance SA results on Twitter data. To analyze sentiment diffusion, the authors first investigated a phenomenon named sentiment reversal and found several exciting characteristics associated with sentiment reversals. Then they considered the associations among textual information in Twitter messages and diffusion patterns of sentiment for predicting sentiment polarities from Twitter messages. Extensive experiments on a real-world dataset show that the proposed approach improves the region beneath the Precision-Recall curve on the Twitter sentiment classification task when compared to state-of-the-art textual information-based SA techniques. Hao et al. [17] proposed a unique approach called CrossWord that handles cross-domain sentiment encoding problems using the stochastic word embedding technique. The proposed method provides an improved way of predicting probabilistic similarity associations between pivot words and the words in the source domain, in addition to labeled reviews in the source domain and unlabeled reviews in both domains.

SentiVec, a kernel optimization method for sentiment word embedding, is suggested by Zhu et al. [53]. The first phase of this study involves supervised learning, whereas the second phase involves unsupervised updating models like object-word-to-surrounding-word reward models (O2SR) and context-to-object-word reward models (C2OR). Experimental findings demonstrate that the optimum sentiment vectors effectively retrieve the features in terms of semantics and sentiment analysis, outperforming baseline approaches on tasks such as word analogy, similarity, and sentiment analysis. Bidirectional Encoder Representations from Transformers (BERT) model is employed to classify the public thoughts on Covid-19 by Singh et al. [48]. The authors performed sentiment analysis on two data sets in this paper: one data set contains tweets from individuals across the world, while the other data set comprises tweets from individuals in India. The validation accuracy of sentiment categorization is 94 percent, according to the experimental data.

A new method called the sentiment-based rating prediction method is suggested by Munuswamy et al. [36] to develop a recommendation system, which is proficient to mine useful knowledge from the user reviews posted on social media platforms to forecast the exact details adored by users based on their ratings. The opinions of users on an item are calculated using a sentiment dictionary in this model. Furthermore, item reputations are calculated using the three sentiments to anticipate and produce correct suggestions. For efficient classification of reviews posted on social media platforms, the n-gram methodology is included as a contemporary feature in semantic analysis and syntax, together with SVM, to boost the accuracy of the results.

A variety of classifiers and feature sets to perform sentiment quantification are explored by Ayyub et al. [6]. Based on the features set, an empirical performance assessment of traditional machine learning-based approaches, ensemble-based methods, and state-of-the-art deep learning-based methods is undertaken. The results reveal that different feature sets have an impact on classifier performance in sentiment quantification. The findings also demonstrate that deep learning methods outperform traditional machine learning algorithms. The analysis of 104 mental health apps on the App Store and Google Play with the help of five supervised machine learning techniques to perform sentiment classification on 88,125 user reviews is performed by Oyebode et al. [38] The top-performing classifier was then employed to forecast the sentiment polarity of reviews. Then, using the thematic assessment of negative and positive reviews, the authors found themes that represent numerous elements that influence the success of mental health apps in both positive and negative ways.

A unified framework that helps in bridging the gap between machine learning-based approaches and lexicon-based approaches is presented by Iqbal and Hashmi [15]. The authors developed a unique Genetic Algorithm-based feature reduction technique for addressing the scalability issue that occurs as the feature set develops. The authors successfully minimized the size of the feature set by 42% while maintaining accuracy by utilizing the proposed hybrid approach. Khan and Gul [22] used the Bag of Words (BoW) technique for obtaining features from movie reviews posted online & expressed those features as a vector. Then, the authors employed the Naive Bayes machine learning algorithm to categorize movie reviews expressed in the form of feature vectors into positive & negative classes. After that using the pairwise semantic similarities between categorized review sentences, an undirected weighted network was created, with the graph nodes representing review sentences and graph edges indicating semantic similarity weight. The absolute measure for all the review sentences in the graph was computed using the weighted graph-based ranking algorithm (WGRA). In the end, the extracted summary was created by selecting the top-rated sentences (graph nodes) built on the highest-ranking measures.

Chiong et al. [11] proposed 90 unique features that can be fed to a machine learning classifier to detect depression by analyzing users’ social media posts. The authors used a combination of sentiment lexicons and content-based approaches to extract these features. A comprehensive study of these features was conducted using two datasets of Twitter posts, four single classifiers, and four ensemble models. The authors found that all the proposed depression detection features performed best when used together, but the effectiveness of different feature groups ranged greatly. Additionally, the authors also found that ensemble models are more capable of overcoming data imbalance than single classifiers.

### Aspect based sentiment analysis (ABSA) techniques

As a robust method for enhancing sentiment analysis accuracy, aspect terms extraction has attracted significant attention from researchers. This section discusses the numerous studies that have been recently suggested on ABSA [2, 3, 8, 21, 37, 41, 44, 46, 50].

Schouten et al. [44] presented a text-handling framework that can condense reviews. The subtask of this framework is to discover the general aspect types referred to in review sentences, for which the authors proposed two approaches in this paper. The first approach proposed is an unsupervised procedure that finds aspect categories by applying association rule mining to co-occurrence frequency data gathered from a corpus. With an F1-measure of 67 percent, the proposed unsupervised technique outperforms numerous simple baselines. The second technique proposed by the authors is a supervised variation that is found to perform better than the existing methods with an F1-measure of 84 percent. An ABSA hybrid approach to analyze the entities of smart app reviews integrating domain lexicons and rules is presented by Alqaryouti et al. [2]. In this method, language processing techniques, rules, and lexicons are utilized to address several challenges of sentiment analysis, and summarized results are produced. When implicit aspects are considered, the aspect extraction accuracy is found to improve dramatically.

Wang et al. [50] proposed a coarse alignment mechanism called Cross-Lingual Sentiment Classification to make an aspect-level fine-grained system from a group-to-group topic alignment. This unsupervised aspect, opinion, and sentiment unification model (AOS), trimodels aspects, opinions, and sentiments of reviews from various fields and uses a coarse alignment approach to help obtain more precise latent feature representation. To improve AOS even more, the authors offered ps-AOS, a partially supervised AOS model in which labeled source language data is used to reduce the difference in feature representations across two language domains via logistics regression. The results of massive experiments conducted on different multilingual product review datasets reveal that ps-AOS substantially outstrips several state-of-the-art methods. To optimize aspect-based sentiment analysis, one of the leading challenges of bipolar words in SA is addressed by Nandal et al. [37]. This research study examined the words changing polarity in the presence of context, its impact on the overall rating of the product, as well as the particular aspect, and came up with impressive results.

An automatic method is presented by Parthi et al. [41] to calculate sentiments of dynamic aspects from customer-generated reviews gathered through multiple sources using web scraping to deal with the cold start issue. By appending new stop words to the system, the authors improved the accuracy of the system. To address the issues of cost and time complexity, an unsupervised learning-centered approach for ABSA is recommended by Shams et al. [46]. The approach is based on three coarse-grained stages that are split into several fine-grained operations. The preliminary polarity lexicon and aspect word sets, as representations of aspects, are selected in the first stage to extract preceding domain knowledge from the dataset. As primitive information, these two resources are fed into an expectation–maximization algorithm, which determines the possibility of any word based on its aspect and emotion. To identify the polarity of any aspect in the last stage, the document is first split into its constituent aspects, and the possibility of each aspect/polarity is determined based on the document.

The end-to-end ABSA is investigated by Bie and Yang [8], and a unique multitask multiview network (MTMVN) model is proposed. The model prioritizes the unified ABSA as the primary job, with the two sub-chores: aspect term mining and aspect sentiment prediction as auxiliary chores. The authors considered the information gathered from the branch network of the central chore as the global view, and the information gathered from the two sub-chores as the two local views, each with diverse eminences. A multitasking approach enables crucial task completion through the incorporation of aspect boundary data and opinion polarity data. A Joint Aspect-level Sentiment Modification (JASM) model proposed by Jiang et al. [21] addresses the problem of reversing sentiment without affecting sentiments of other aspects within a sentence. With JASM, two coupled modules are jointly trained: aspect-specific sentiment word extraction and aspect-level sentiment transformation. In addition, the authors proposed a mechanism for learning aspect-aware sentiment representations and a method for dynamically selecting the next words based on aspect-aware sentiments or content information.

Aurangzeb et al. [3] presented a novel ABSA technique called Evolutionary Ensembler (EEn) to improve the diversity and accuracy of multi-label classifiers. The ABSA-based EEn highlighted multi-label-based models' accuracy and variety. To assess the accuracy and variety of learners using machine learning and to produce accurate and diverse forecasts, EEn dynamically optimizes two objective functions using an evolutionary multi-objective optimization technique.

### Deep learning-based SA methods

Deep learning-based methods have also been applied to the sentiment analysis domain recently to further improve efficiency [1, 4, 5, 10, 12, 14, 20, 25, 26, 29, 30, 52].

The first end-to-end SEmi-supervised Multi-task Learning framework (SEML) for performing ABSA on user reviews is presented by Li et al. [26]. Both aspect mining and aspect sentiment classification in ABSA is learned together in a joint session. SEML also uses Moving-window Attentive Gated Recurrent Units (MAGRU) to create combined representations of reviews from three stacked and bidirectional neural layers. MAGRU expands GRU with the moving-window attention mechanism for obtaining substantial surrounding semantic contexts. The proposed model also uses Cross-View Training (CVT) for training auxiliary prediction modules on non-labeled reviews for enhancing representation learning.

The aspect-based sentiment analysis for demonetization tweets is performed using the improved deep learning method by Datta and Chakrabarti [12]. Pre-processing, extraction of aspects, polarity features, and sentiment categorization are all phases of the proposed model. To begin, the various demonetization tweets from the Kaggle data set are captured and pre-processed. Aspect extraction is used for the extraction of the sentiment words from the pre-processed data. With the support of polarity measure calculation and Word2vec, these retrieved aspect words are transformed into features. The authors optimized the polarity measures by integrating FireFly Algorithm (FF), and Multi-Verse Optimization (MVO), producing a novel algorithm called FireFly-oriented Multi-Verse Optimizer (FF-MVO). Recurrent Neural Network (RNN) is then applied to the combined features for the classification of sentiments as positive and negative.

Kumar et al. [25] suggested a method for sentiment analysis that combines three methods: developing ontologies for extracting semantic features, using Word2vec for transforming processed corpora, and creating convolutional neural networks (CNNs) for mining opinions. Particle swarm optimization is used for CNN parameter tuning to find non-dominant Pareto front optimal values. Using both ML and deep learning methods, an aspect-based sentiment analysis approach using polarity classification and sentiment extraction on reviews is recommended by Alamanda [1] to automatically extract the most interesting polarity aspects desired by customers. A search engine is being created to pull up tweets and reviews relating to a user-specified phrase and display the accompanying noteworthy aspects.

An aspect-gated graph convolutional network (AGGCN) is proposed by Lu et al. [30], which comprises a special aspect gate for guiding the encryption of aspect-specific information and employs a graph convolution network based on the sentence dependency tree for fully exploiting syntactical information and sentiment information. The model fails to address the issues related to noise and biases that get introduced during the encoding of aspect-specific information. Dragoni et al. [14] proposed an enhanced version of OntoSenticNet, a conceptual model for structuring emotions from multimodal resources, with SenticNet as a commonsense knowledge base for sentiment analysis. In addition to supporting the execution of semantic sentiment operations at reasoning time, the model also supports a conceptual model for sentiment dependencies and discovery paths.

Huang et al. [20] introduced a unique model for text sentiment recognition called Attention Emotion Enhanced (AEC)-LSTM, which seeks to improve the LSTM network by incorporating attention mechanism and emotional intelligence. First, an emotion-enhanced LSTM network is proposed, referred to as ELSTM, in which emotion modulation of learning is achieved using an emotion modulator and emotion estimator to enhance the feature learning ability of LSTM networks. The authors further integrated ELSTM with other operations, like convolution, pooling, and concatenation, to provide a better representation of various structure patterns in text sequences. Then, using the topic-level attention mechanism weight of hidden representations of text is adaptively adjusted. A Broad Multitask Transformer Network (BMT-Net) proposed by Zhang et al. [52] combines a feature-based approach with fine-tuning approach. The purpose of this system is to explore the high-level information of robust and contextual representations. The proposed structure enables global representations to be learned across tasks using multitask transformers. As a result of its ability to search for suitable features in deep and broad ways, BMT-Net can roundly learn the robust contextual representation that is used by the broad learning system.

Aygun et al. [5] employed ABSA technique to determine the attitude of Twitter users from the countries like Canada, France, Turkey, UK, USA, Spain, Italy, and Germany towards vaccination and vaccine types during the COVID-19 period. In this study, two datasets were prepared (English and Turkish language), containing 928,402 tweets related to vaccines. The authors used four different BERT models (mBERT-base, BioBERT, ClinicalBERT, and BERTurk) to classify tweets on four different aspects (policy, health, media, and others). Six different COVID-19 vaccines selected from the datasets were subjected to sentiment analysis using Twitter posts related to these vaccines. The authors obtained a total accuracy of 87% to present the views of the users on Covid-19 vaccination. The work proposed by Chandra et al. [10] compares selected translations of the Bhagavad Gita (mostly from Sanskrit to English) using semantic and sentiment analysis. The authors employed a hand-labeled sentiment dataset to fine-tune BERT model. The semantic analysis of selected chapters and verses across translations was also provided using novel sentence embedding models.

Based on tweets related to cryptocurrency, the work proposed by Aslam et al. [4] performs sentiment analysis as well as emotion detection for forecasting cryptocurrency market value. To increase the efficiency of the analysis, LSTM-GRU is proposed, a deep learning ensemble model that combines the features of two different RNN applications, including LSTM and Gated Recurrent Unit (GRU). Londhe et al. [29] suggested a novel method for ABSA utilizing the deep learning classifier LSTM-RNN. The hybrid LSTM-RNN is found to analyze and forecast the polarity of aspects with the highest degree of accuracy. Additionally, the major output is the correct extraction of multiple aspects from lengthy reviews with many sentences.

### Motivation

Sentiment analysis has recently received substantial attention from researchers for a variety of applications, hence a framework to perform it efficiently while addressing scalability is needed. We investigated a variety of strategies employed to perform sentiment analysis, intended to improve the feature representation and classification using machine learning/deep learning-based techniques. The inclusion of fraudulent, sarcasm, spam, emoticons, negation and other types of content in a significant number of online reviews makes feature extraction challenging. The study carried out in this section shows that deep learning methods are more efficient than machine learning methods for sentiment classification. The accuracy of deep learning algorithms, we believe, is mostly determined by the feature set.

According to the findings of the studies mentioned above, sentiment analysis-based approaches are insufficient to handle the issues of accurately representing sentiments, opinions, and emotions, from online reviews. As a result, the ABSA approaches recently proposed are studied, however, their scope or application is also limited as they address the challenge of ambiguity and sarcasm to some extent, but don't consider emotions, negations, and long-term dependencies for accurate classification.

Aside from these issues, scalable review datasets have not been used to evaluate ABSA approaches. Some ABSA approaches relied primarily on unsupervised procedures, necessitating a significant number of manual data annotations. Because some SA/ABSA algorithms rely solely on symbolic feature extraction, their accuracy suffers. In comparison to machine learning and lexicon-based techniques, deep learning-based techniques provided superior scalability and efficiency.

### Contribution

We suggest a novel technique of SA with special reference to consumer review summarization, based on the above-mentioned concerns of the state-of-the-art approaches. The suggested method comprises three stages: pre-processing, feature extraction, and sentiment classification. To denoise input reviews, the pre-processing step employs a standard procedure. The feature extraction step is ingeniously built as a hybrid step to overcome the difficulties of accurately representing the features of input reviews. In particular, the proposed model has the following contributions:

- Based on our observations that hybrid feature sets accomplish effective results, we split the feature extraction phase into two parts: Review-Related Features (RRF) and Aspect-Related Features (ARF). After that, RRF and ARF numerical features are merged into a Hybrid Feature Vector (HFV).
- As part of RRF, various methods like term frequency-inverse document frequency (TF-IDF), n-gram features & emoticon polarities, are employed for making emotional representations more accurate.
- As part of ARF, we propose a novel way for extracting aspects terms employing co-occurrence frequencies and then assigning the polarities. The ARF method produces aspect terms with their polarities.
- For the classification task, a deep learning classifier LSTM is used for the classification of input reviews either into negative, positive, or neutral classes.

## Proposed system

According to the findings of the aforementioned studies, sentiment analysis has become a popular study area due to several intriguing and challenging research topics. An enormous number of start-up organizations are aiming to provide sentiment analysis/review mining services due to their diverse functional uses. Every firm wants to know how its products and services are perceived by its customers. This domain will remain active and dynamic in the coming years due to scientific challenges and functional components. The goal of this work is to develop a hybrid model to perform sentiment analysis accurately from the perspective of consumer review summarization.

**Fig. 1:**
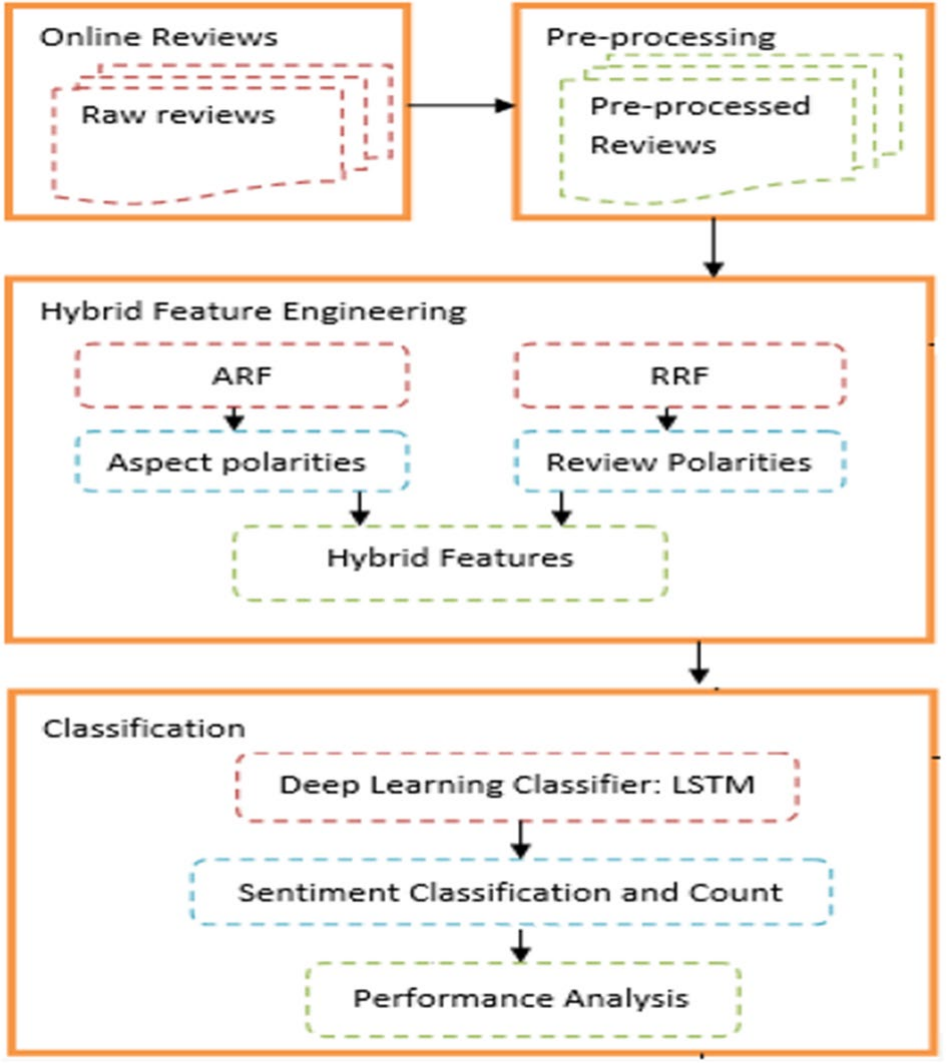
Proposed architecture for SA using HFV + LSTM

The different steps followed in this study are shown in Fig. 1. The initial step comprises the data collection & pre-processing of data. In the second step, feature extraction is done using the RRF and ARF techniques and then, RRF and ARF numerical features are merged into a hybrid feature vector-HFV. A deep learning classifier LSTM uses the HFV to classify the input reviews into positive, negative, or neutral categories. Then the performance of the model is compared using certain metrics. The subsections below explain every step of Fig. 1.

### Data collection and pre-processing

The SemEval-2014 restaurant reviews data set [32], Sentiment140 [16], and STS-Gold [42] datasets have been employed to assess the proposed model and compare it to the recent state-of-the-art methods. The SemEval-2014 restaurant reviews dataset comprises 3000 training reviews and 800 testing input reviews. All the reviews in this data set have at least one annotated aspect term. The Sentiment140 is a Twitter dataset with 1.6 million annotated tweets (0 being negative, 2 being neutral, 4 being positive). The STS-Gold dataset includes 2026 tweets with their IDs and polarities.

The pre-processing of the dataset consisting of raw reviews is done by deleting & fixing the intricate and pointless text. The data pre-processing functionality used in the proposed work is beginning with tokenization and finishes with the removal of meaningless words, digits, and words less than three characters. Using the tokenization function, we subdivided the input reviews into tokens. Then we applied stemming to each token to cut it down to its singular form (e.g., performed or performing gets reduced to perform). Then, to limit the number of tokens, stop-words like ‘I’, 'a', 'an', 'am', and so on are deleted. Dates, special characters like #, @, etc., URLs, and meaningless words (b+, C−, etc.) are all found and eliminated. Furthermore, words with less than three letters and integers are also found and eliminated. This algorithm assures that the dimensional space of raw reviews is effectively reduced. Table 1 below shows some sample reviews before pre-processing and after pre-processing.

**Table 1:** Sample reviews before and after employing pre-processing algorithm

Before pre-processing	After pre-processing
“The Pad seew chicken was delicious, however, pad thai was more than usually greasy”	“Pad chicken delicious pad thai greasy”
“I was very disappointed with this restaurant”	“disappoint restaurant”
“I had to ask cart attendant three times before she finally came back with the dish lotus leaf wrapped rice that I’ve requested.”	“ask cart attendant three time come back lotus leaf wrap rice request”
“Food was fine, nothing great”	“Food fine nothing great”

### Hybrid feature vector

There have been several attempts to represent input reviews as features. Precise, robust, & effective feature extraction, still, remains a difficult research challenge for review mining. As a result, developing an efficient and consistent feature set that can yield high categorization accuracy is an important aspect of any sentiment analysis system. Therefore, we propose a robust and effective sentiment analysis architecture employing hybrid feature extraction methodology. In the first step, the extraction of RRF features is done by employing various methods to achieve the polarity for every term within the preprocessed text including negations and emoticons. The aspect terms with their polarities are then extracted using the ARF method. Sarcastic and ambiguous reviews are also addressed and represented by the ARF. Finally, by integrating the results of ARF and RRF, every preprocessed review is expressed as HFV.

#### Review related features (RRF)

RRF is a sentence-level feature representation strategy that considers emoticons as well along with text to describe feelings, opinions, and negations in the input reviews. To construct the hybrid form of features, traditional approaches like n-gram, TF-IDF, & emoticons-specific features have been combined. The TF-IDF approach uses BoWs, and the n-gram relies on word embeddings. The usage of solitary words for extracting features limits sentiment analysis in various ways. The negation problems don’t get solved with a single-word feature, and at the same time, it can contribute to the inappropriate classification of sentiments.

We addressed these issues first by obtaining a word list from the pre-processed reviews by n-gram feature extraction. The TF-IDF of n-gram words is then calculated using the TF-IDF on the n-gram yield. The combination of n-gram and TF-IDF technique is not just shrinking the dimensionality but resulting in an effective representation of each review as well. The emoticons-specific features are then retrieved for improving the sentiment analysis accuracy further. As a result, RRF is attained as a combination of n-gram, TF-IDF, & emoticons-specific features. The RRF procedure is described in detail below.

Let us assume that Q^i^ is the document comprising the ith review that has been pre-processed. We start with the application of the n-gram approach on a preprocessed document for blended n-gram and TF-IDF. An n-gram is a proximal series of n-words obtained from a given dataset. An n-gram model with n = 1 is known as a unigram, when n is 2, it is referred to as a bigram, when n is 3, it is referred to as a trigram, and so on. "Bad" and "Very Bad," for example, are unigrams and bigrams, correspondingly. From the input sentence, the n-gram approach constructs a sequence of n consecutive words as follows:

1 $$Ngram=getNgram({P}^{i}, n)$$

where ${\mathrm{Ngram}}^{\mathrm{i}}$ *Ngram*^*i*^ denotes the set of n-grams derived from the preprocessed input document ${Q}^{i}$. The parameters ${Q}^{i}$ and n are passed to the function ${\mathrm{Ngram}}^{\mathrm{i}}$ *Ngram*^*d*^ We fixed the value of $\mathrm{n}$ n to 2 in this approach to strike a balance between efficiency and accuracy when dealing with negations.

Following the results of the n-gram, TF-IDF is employed for getting the inverse document frequency for the word lists resulting from the training/testing datasets. Term Frequency (TF) indicates how repeatedly a term exists throughout the document and Inverse Document Frequency (IDF) is a measure that finds out if a term is unusual or common among all the documents in a corpus.

2 $$TF-IDF= TF\left( {Ngram}^{i}\right)\times IDF({Ngram}^{d})$$

where ${\mathrm{Ngram}}^{\mathrm{i}}$ *Ngram*^*i*^ signifies the list of words associated with the ith ith review and ${\mathrm{Ngram}}^{\mathrm{i}}$ *Ngram*^*d*^ signifies the list of words associated with the complete data set $\mathrm{d}$ d. For instance, assume *Ngram*^*i*^ consists of 60 terms and the term “good” occurs 5 times, the result of TF is $\frac{5}{60}=0.08$. Likewise, if a document has 600 words and the term “good” occurs 60 times, the IDF is calculated as $\mathrm{log}\left(\frac{600}{60}\right)=1$. Using (2), the TF-IDF is calculated as $0.08 \times 1=0.08$ 0.08*1 = 0.08 for the term “good” in the ith review. A vector NT(i) is then generated for each feature within the document:

3 $$NT\left(i\right)= Ngram+TF-IDF$$

From then on, the emoticons-specific features are then retrieved from the review dataset and represented as a vector for categorization and subsequent computations. Because every review may or may not contain emoticons, we set the value of the emoticons feature vector (EF) of size 1 × 2 to zero for each review. The discrete probability distribution formula is used to count the emoticons for every review alongside the sentiment label. A positive emoji is represented as 1, while a negative emoji is represented as − 1. Suppose the review comprises of six emoticons, three positive and three negative, then the outcome will be represented as [3, − 3]. When positive, negative, or both emoticons are missing from a review, the emoticons-related features are given a value of zero. The emoticons-specific features are then joined with $\mathrm{NT}$ features, resulting in the rewrite of Eq. (3) as:

4 $$NTE\left(i\right)=[NT, EF]$$

#### Aspect related features (ARF)

We apply the ARF extraction procedure to training and testing datasets after the extraction of the hybrid version of review-specific features, which uses the mechanism derived from Schouten et al. [44] solely to boost sentiment analysis results. The goal is to count: lemmas' co-occurrences with sentence annotated categories, lemmas' co-occurrences with aspect types, and grammatical dependencies' co-occurrences with aspect types. In this section, we rebuild the ARF approach by approximating the weight matrix corresponding to every category set, which is then translated into aspect features for every preprocessed review in the input data set. In this study, we bypassed the category valuation technique and instead concentrated on the extraction of the aspect terms from every review together with their co-occurrence frequencies. As opposed to Schouten et al. [44], it saves significant amounts of computation time of employing the supervised classification algorithm.

The suggested ARF method is demonstrated in Algorithm I. Let Q denote the training set, which consists of m raw internet reviews. In each input review, we performed the extraction of the categories followed by the estimation of their co-occurrence rates against the lemmas and dependencies forms. The ARF algorithm's initial step is to identify each review's set of lemmas, dependencies, & categories. The lemma represents the dictionary form of a word, while dependencies show the binary grammatical relationship existing between a term called governor or head and another term called modifier or dependent in a sentence.

The instance below demonstrates a review comprising dependency relations, with the words 'price' (governor) and 'okay' (dependent) making a dependency relation named nsubj (subject-predicate relation), and the words 'very' (governor) and 'attractive' (dependent) making one more dependency relation termed avdmod (adverbial clause modifier).
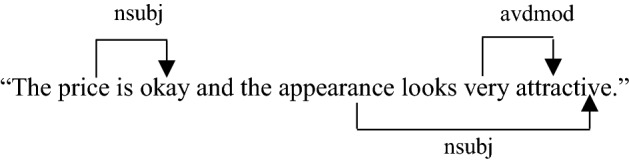


The list of lemmas and dependence forms are contained in set *S*. For input review, the ${\mathrm{s}}_{\mathrm{C}}$ *S*_*C*_ comprises a list of aspect categories. Each review is handled using the Standford CoreNLP framework's dependency parser, POS tagger, and lemmatizer functions as an NLP process [42]. The subsequent action is to count and add the lemma or dependency form's unique occurrences to vector $\mathrm{Y}$ Y. Likewise, all input review aspect categories are detected and added to vector $\mathrm{C}$ C. We save the co-occurrence frequency in vector $\mathrm{X}$ after detecting the lemma/form dependency and unique categories. Furthermore, the occurrence frequencies for all the lemmas and dependency forms of the analogous reviews are logged into the $\mathrm{vector Y}$.

Based on co-occurrence and occurrence frequency values in vectors $\mathrm{X and Y}$ respectively, the weight matrix is calculated for every pair of $\mathrm{vector X}$ and stored in $\mathrm{vector W}$. A weight frequency value is computed for every pair in $\mathrm{X}$ X only if the analogous co-occurrence frequency exceeds zero. This method eliminates the challenge of finding the optimal threshold in any dataset. Our final step in estimating the aspect-specific features is to take the highest co-occurrence value for each pair of weights in vector $\mathrm{W}$ W into vector $\mathrm{A}$ A. Using this approach, the goal of extraction of the aspect-related features without high processing constraints is achieved.
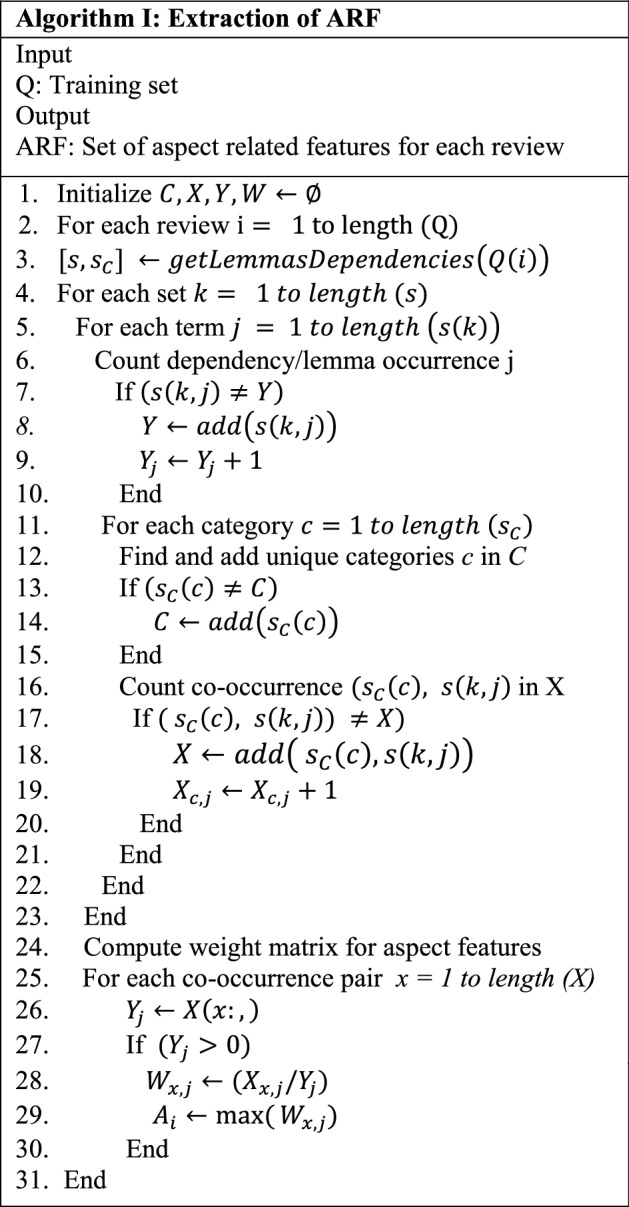


**Fig. 2:**
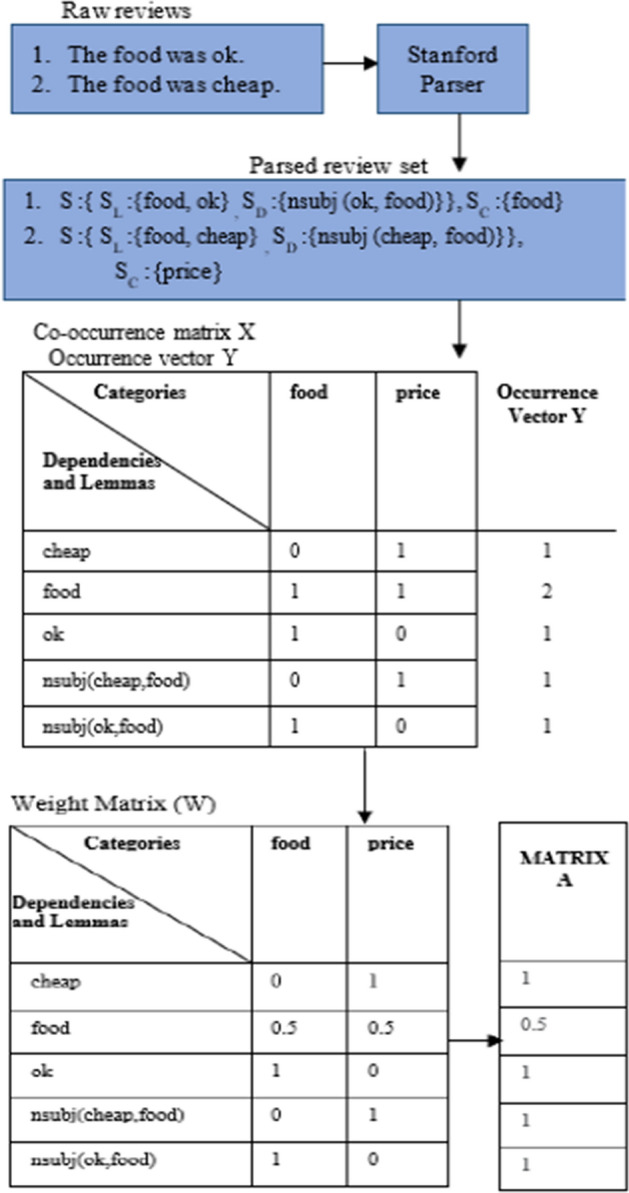
Explanation of ARF

The explanation of Algorithm I is given in Fig. 2 with the help of an example.

### Classification of Sentiments using LSTM

As demonstrated in Fig. 2, hybrid feature representation has been achieved through the combination of ARF and RRF to form a hybrid feature vector-HFV. For the classification of the input hybrid features vector into positive, negative, or neutral sentiment, we have employed the Long-Term Short Term (LSTM) classifier.

The primary issue with using neural network classifiers and conventional classifiers is the vanishing gradient or exploding gradient. The vanishing gradient problem hinders the learning of long data sequences. As a result of the exploding gradient problem, the weights oscillate, further degrading the network quality. Therefore, when neural networks like CNNs/RNNs attempt to store information over extended time intervals, it takes prolonged time due to inadequate, crumbling error backflow. LSTM network, on the other hand, is a kind of RNN proficient in learning order dependence in sequence prediction problems. To overcome the vanishing gradient problem, LSTM uses a distinctive additive gradient structure that incorporates direct access to the forget gates' activations at every time step of the learning process. Thus, it defeats the error backflow problems with the minimum computational complexity of O(1). In the proposed work, we use LSTM for the automatic sentiment analysis from the sequential features vector received for each sequential review. It accepts the training dataset and test sample features vector ${\mathbf{H}\mathbf{F}\mathbf{V}}^{\mathbf{i}}$ as input for each *i*th review.

Let’s suppose that the LSTM input layer receives the ${\mathbf{H}\mathbf{F}\mathbf{V}}^{\mathbf{i}}$ at the current time interval t. LSTM network comprises a memory cell c, an input gate i, an output gate o, and a forget gate f [35]. In every instance of time, LSTM computations consist of computing gate activations {${\mathbf{i}}_{\mathbf{t}}$, ${\mathbf{f}}_{\mathbf{t}}$}, updating its memory cell from ${\mathbf{c}}_{\mathbf{t}-1}$ to ${\mathbf{c}}_{\mathbf{t}}$, calculating the output gate activation ${\mathbf{o}}_{\mathbf{t}},$ and outputting the hidden representation $\mathbf{h}$. The hidden representation from the preceding time instance is ${\mathbf{h}}_{\mathbf{t}-1}$. For the update function, the following equations are used in LSTM:

5 $${i}_{t}=\sigma \left({W}_{i}{HFV}^{i} +{U}_{i}{h}_{t-1}+{V}_{i}{c}_{t-1}+{b}_{i}\right)$$

6 $${f}_{t}=\sigma \left({W}_{f}{HFV}^{i} +{U}_{f}{h}_{t-1}+{V}_{i}{c}_{t-1}+{b}_{f}\right)$$

7 $${c}_{t}={f}_{t}\Theta \boldsymbol{ }{c}_{t-1}+{i}_{t} \Theta \mathrm{ tanh }\left({W}_{c}{HFV}^{i} +{U}_{c}{h}_{t-1}+{U}_{c}{h}_{t-1}\right)$$

8 $${o}_{t}=\sigma \left({W}_{o}{HFV}^{i} +{U}_{o}{h}_{t-1}+{V}_{i}{c}_{t-1}+{b}_{o}\right)$$

9 $${h}_{t}={o}_{t}\Theta \mathrm{ tanh }\left({c}_{t}\right)$$

where Θ represents an element-wise product of the output of the fully connected layers, and σ is the logistic function. The tanh activation function keeps the value of new information between − 1 and 1. The weight matrices (W_f_, W_i_, W_o_, W_c_, U_f_, U_i_, U_o_, U_c_) and biases (b_f_, b_i_, b_o_, b_c_) are not time-dependent which means that the outputs of different timesteps are calculated using the same weight matrices. The input and forget gates work together to refresh the memory cell. The forget gate examines the part of memory to be forgotten, while an input gate approximates new values corresponding to the view currently stored in the memory cell. As a result of the output gate and memory cell, the hidden description is estimated. Due to LSTM activation including summation over time and derivatives distributed over sums, the gradient in LSTM gets spread over extended periods and gets vanishes. The fully connected layer followed by the softmax layer works on classifying the input features into appropriate matching classes according to the training dataset. The output layer generates the final sentiment detection outcome.

## Experimental results

We used MATLAB tool, Windows 11 operating system with an i5 processor for investigational evaluation of the proposed work. The SemEval-2014 restaurant reviews dataset, Sentiment140, and STS-Gold datasets have been used to evaluate the proposed model and compare it to the recent state-of-the-art methods. We have divided all three datasets into 70% training and 30% testing datasets. To demonstrate the efficacy of the hybrid feature engineering methodology, HFV performance is first compared to the proposed RRF and ARF methods employing the LSTM classifier using the SemEval-2014 restaurant reviews dataset. In addition, the comparison of the proposed hybrid feature extraction approach with the recent state-of-the-art methods is also done using all three datasets. The different metrics used to evaluate performance of ARF, RRF, and HFV are F1-measure, accuracy, recall, precision, and Mean Sentiment Analysis Time (MSAT). The MSAT represents the average processing time, i.e., the computing time of the sentiment categorization. For the estimation of the MSAT evaluation parameter, we executed 50 instances of sentiment analysis classification.

### Performance Evaluation of ARF, RRF and HFV

**Fig. 3:**
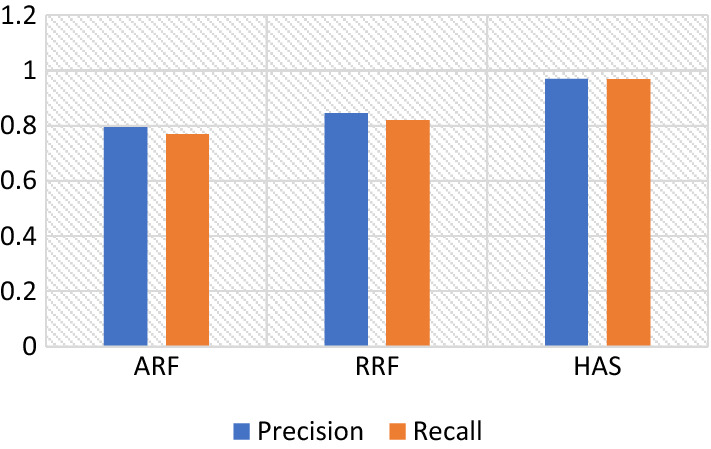
Precision and recall assessment of the proposed feature extraction methodologies using LSTM

**Fig. 4:**
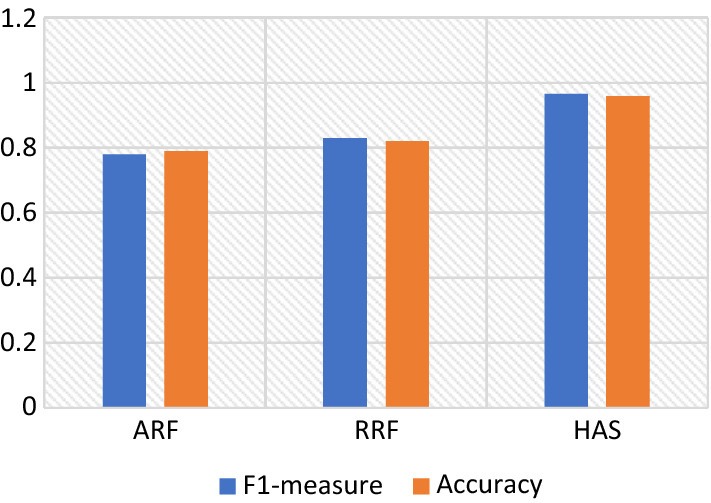
F1-measure and accuracy assessment of the proposed feature extraction methodologies using LSTM

Based on SemEval-2014 restaurant reviews dataset, the performance evaluation of RRF, ARF, and HFV using LSTM is presented in this section Figs. 3 and 4 exhibit the accomplishments of precision, recall, accuracy, and F1-measure, and their accompanying values are presented in Table 2.

**Table 2:** Comparison of proposed features extraction methodologies using LSTM

	F1-Measure	Precision	Recall	Accuracy
ARF	0.779	0.795	0.771	0.789
RRF	0.829	0.845	0.821	0.82
HFV	0.967	0.971	0.969	0.959

It can be observed from the examination of F1-measure, accuracy, precision, and recall that the hybrid feature engineering method gave much better results for sentiment classification than ARF and RRF using LSTM. The HFV strategy encompasses the issues associated with sarcasm, negation, ambiguity, emotions, opinions, and feelings regarding restaurants and their aspects, which is the primary cause of this performance improvement. The combination of n-gram, TF-IDF, and emoticons-specific features results in the attainment of the RRF vector. It successfully constructs the hybrid feature vector, which conveys the frequency of words occurring in every review alongside emoticon-specific features. To further limit sentiment analysis errors, the ARF approach employs the co-occurrence frequency strategy for managing sarcastic and ambiguous phrases. The RRF outperformed the ARF in terms of results because it successfully addressed negations, opinions, and emotions.

**Fig. 5:**
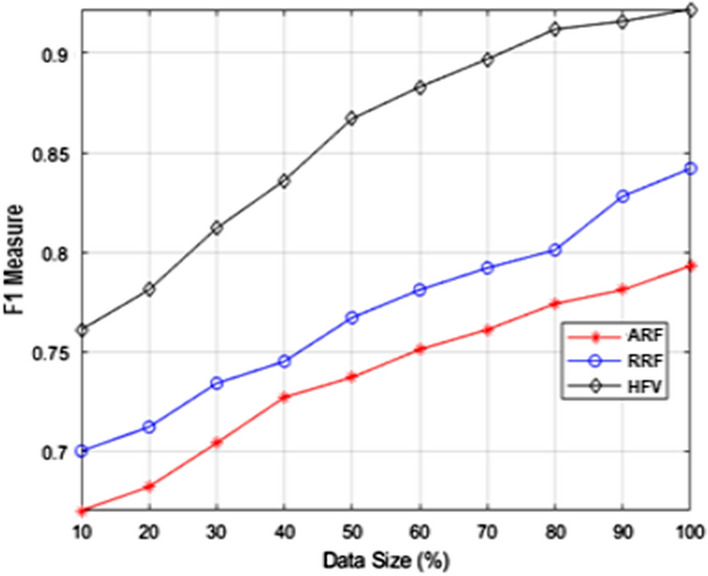
F1-measure examination with altering data size

**Fig. 6:**
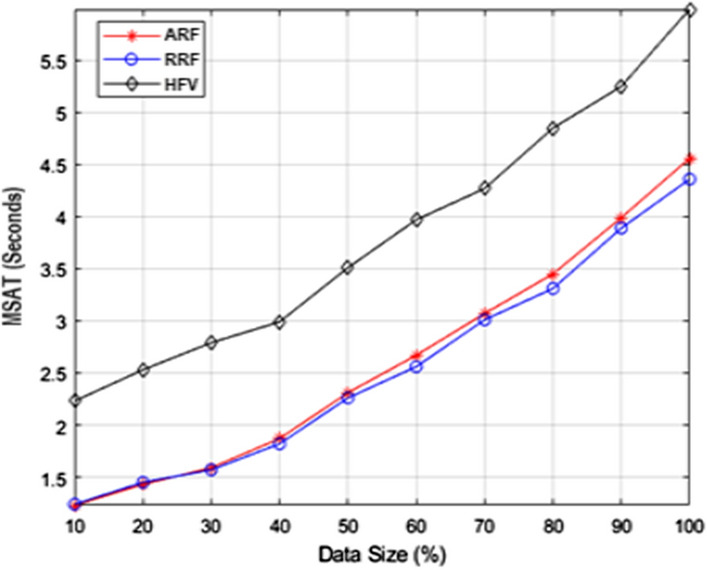
MSAT examination with altering data size

The performance of F1-measure and MSAT with varied training data set sizes is shown in Figs. 5 and 6 respectively. The training dataset was divided into smaller groups of varying sizes using the stratified sampling process. Each iteration results in a 10 percent rise in training data, as illustrated in Figs. 5 and 6. The HFV method outperforms the ARF and RRF techniques in terms of F1-measure performance. Because there is a vast amount of labeled data available for precise prediction, the F1-measure performance improves as the data size grows. According to the MSAT analysis, HFV requires a higher processing time than ARF and RRF feature extraction strategies. Nevertheless, considering the improvements that the hybrid feature vector has shown in sentiment analysis, it is acceptable.

### State-of-the-art investigations

Using the SemEval-2014, STS-Gold and Sentiment140 datasets, we compared the proposed hybrid feature extraction approach employing LSTM with the below-mentioned recent state-of-the-art methodologies for investigating its quantitative representation.

*Supervised ABSA (SABSA)* [44]: This method evaluates the suggested supervised ML-based aspect category prediction model utilizing the co-occurrence strategy to perform sentiment analysis using the SemEval-2014 dataset. We chose the SABSA strategy for comparison with the suggested model since it is intimately related to our ARF approach.

*SentiVec *[53]: The newly presented technique uses a hybrid approach combining unsupervised and supervised ML methods for performing word embedding intended for sentiment analysis.

*N-gram + TF-IDF + SVM* [6]: Our performance analysis also included this recent approach. The development of the hybrid feature vector suggested in this research article is similar to the methodology proposed in this work for extracting RRF features except for the addition of emoticon-specific features to RRF.

*SEML* [26]: It's an aspect-based sentiment analysis technique that uses a semi-supervised classifier to perform aspect mining and sentiment classification. They also analyzed performance using the SemEval-2014 dataset.

*MTMVN* [8]: Since it is a newly proposed method for aspect-based sentiment analysis, it has been selected for comparison with the proposed method. A deep learning classifier is used after the extraction of the aspect-specific features in this paper.

Tables 3, 4, 5 show the comparison of all the above-mentioned approaches based on F1-measure, recall, accuracy, precision, and Area Under Curve (AUC) evaluation parameters employing the SemEval-2014 restaurant review dataset, STS-Gold dataset and Sentiment140 dataset. For all three datasets, the suggested hybrid feature extraction approach employing LSTM (HFV + LSTM) shows enhanced performance for all the considered evaluation parameters. The enhanced performance is due to its capacity to create the hybrid feature vector utilizing several strategies robustly and efficiently. The dataset Sentiment140 delivered a higher F1-score, precision, recall, accuracy, and AUC performances compared to other datasets SemEval-2014 and STS-Gold. This improvement is because there are around 1,60,000 reviews in the Sentiment140 dataset, which is much larger than the other two datasets. It enables more accurate classifications using each classifier due to the large number of samples for each class in the dataset. The STS-Gold delivered lower efficiency using each classifier than the SemEval-2014 and Sentiment140 datasets as it has fewer training samples.

**Table 3:** Comparison of HFV + LSTM Model using SemEval-2014 data set with State-of-the-Art Methods

ABSA/SA Technique	Precision	Recall	F1-measure	Accuracy	AUC
SABSA	0.845	0.832	0.839	0.829	0.816
SentiVec	0.863	0.843	0.855	0.847	0.834
Ngram + TF-IDF + SVM	0.851	0.838	0.845	0.837	0.823
SEML	0.841	0.824	0.833	0.826	0.813
MTMVN	0.793	0.773	0.785	0.777	0.765
HFV + LSTM	0.946	0.912	0.922	0.902	0.918

**Table 4:** Comparison of HFV + LSTM Model using Sentiment140 data set with State-of-the-Art Methods

ABSA/SA Technique	Precision	Recall	F1-measure	Accuracy	AUC
SABSA	0.858	0.839	0.8485	0.837	0.830
SentiVec	0.877	0.858	0.8675	0.861	0.849
Ngram + TF-IDF + SVM	0.866	0.846	0.856	0.844	0.838
SEML	0.854	0.837	0.8455	0.838	0.826
MTMVN	0.817	0.789	0.803	0.792	0.789
HFV + LSTM	0.961	0.938	0.9495	0.927	0.933

**Table 5:** Comparison of HFV + LSTM Model using STS-Gold data set with State-of-the-Art Methods

ABSA/SA Technique	Precision	Recall	F1-measure	Accuracy	AUC
SABSA	0.791	0.784	0.7875	0.782	0.763
SentiVec	0.845	0.837	0.841	0.841	0.817
Ngram + TF-IDF + SVM	0.831	0.817	0.824	0.815	0.803
SEML	0.826	0.809	0.8175	0.811	0.798
MTMVN	0.776	0.758	0.767	0.762	0.748
HFV + LSTM	0.927	0.899	0.913	0.900	0.899

In comparison to SentiVec and N-gram + TF-IDF + SVM, the ABSA methods: SABSA, SEML, and MTMVN are found to exhibit poor performance since they lack negation handling and do not extract review-specific features. The model presented in this paper focuses on delivering an integrated solution that combines the advantages of both aspect-specific and review-specific features to increase performance.

## Conclusion and future work

The paper aims to accurately perform sentiment analysis from the point of view of the consumer review summarization model for capitalists. We outlined several research concerns and possible solutions for the challenges that occur when performing sentiment analysis for raw online reviews. Using the hybrid feature extraction method proposed in this paper, the input pre-processed reviews can be transformed into meaningful feature vectors, allowing efficient, reliable, and robust sentiment analysis to take place. The results reveal that as compared to individual methodologies; the hybrid approach greatly improves sentiment analysis performance. We have also compared the proposed model’s performance with the recent state-of-the-art methods for F-1 measure, accuracy, precision, recall, and AUC evaluation parameters. All five evaluation parameters are found to improve significantly. The HFV + LSTM model's future directions include (1) applying sentiment classification results to extend the proposed model for consumer review summarization, (2) investigating the proposed model's performance using diverse datasets, and (3) exploring more NLP techniques to make the model language independent.

## Data Availability

The data used to support the finding of this study are available from the corresponding author upon request.
